# Development of Hot Trub and Coffee Silverskin Phytoextracts for Sustainable Aerosol Disinfectant Application

**DOI:** 10.3390/foods14142496

**Published:** 2025-07-16

**Authors:** James Ziemah, Matthias S. Ullrich, Nikolai Kuhnert

**Affiliations:** School of Science, Constructor University Bremen, 28759 Bremen, Germany; mullrich@constructor.university

**Keywords:** food production waste, log reduction, sustainability, hygiene, bio-disinfectant, chalcones, chemometrics and CGAs

## Abstract

Chemical products, including cleaning agents, disinfectants, stain removers, and cosmetics, release harmful chemicals that pose a risk to human health and the environment, necessitating alternative sources. The objective of this research was to identify the most effective phytoextract from food production waste for use in sustainable aerosol hygiene technology as an electrostatic bio-disinfectant. The investigation was performed through wipe tests and airborne microbial collection techniques. The upgraded coffee silverskin phytoextract demonstrated superior disinfection potential for various surfaces and airborne microbes compared to the hot trub phytoextract, with an industrial disinfectant serving as the control. Log reduction analyses revealed a more significant killing efficacy (*p* ≤ 0.05, using the ANOVA test) against Gram-positive organisms (*Bacillus subtilis* and *Listeria monocytogenes*) than against Gram-negative organisms (*Escherichia coli* and *Vibrio parahaemolyticus*), with the log reductions ranging from 3.08 to 5.56 and 3.72 to 5.81, respectively. Chemical characterization by LC-ESI-QTOF-MS, ^1^H NMR, and FTIR showed that CGAs and chalcones are the most bioactive compounds in CSS and HT, respectively. The innovation in this work involves an integrated approach that combines waste-derived phytoextracts, advanced chemical profiling, and scalable aerosol disinfection. Furthermore, this research offers a greener, cost-effective, and industrially relevant alternative to synthetic chemical disinfectants. The interdisciplinary approach contributes to the development of bio-based disinfectants for use in the food industry, hospitals, and public health settings. This investigation supports a paradigm shift toward sustainable disinfection practices, thereby improving food and environmental safety.

## 1. Introduction

The use of food production waste as a source of bioactive materials highlights a growing interest in circular economy practices and sustainable biotechnology [1]. Rather than treating food production waste as disposable, this approach recognizes its potential for transformation into value-added products, such as antimicrobial agents [2]. This concept also aligns with global sustainability goals and promotes more efficient use of agricultural byproducts as resources [3,4].

Historically, the development of disinfection technologies has been pivotal to reducing infection and improving public health. From the early innovations of Lister and Semmelweis in terms of chemical antiseptics to modern sterilization methods, hygiene practices have continually evolved to address microbial threats [5,6]. Innovations in aerosol-based disinfection using food production waste phytoextract mark the latest step in this progression.

Among these advancements, electrostatic aerosol spraying stands out as a promising technique for broad, efficient microbial control. It enhances the distribution of antimicrobial agents on surfaces and in the air, allowing uniform coverage and reducing the amount of disinfectant needed [7]. This method also offers operational advantages, such as lower labor costs and reduced human exposure to chemical agents [7,8].

Growing concerns over the environmental and health risks associated with synthetic and corrosive disinfectants have created an urgent need for greener alternatives. Bio-based disinfectants derived from natural sources, particularly food industry byproducts, offer a non-toxic, biodegradable, and potentially safer solution for hygiene technologies in public and industrial settings [9,10,11].

Coffee silverskin (CSS) and brewery hot trub (HT) are two abundant food industry byproducts that are rich in secondary plant metabolites, such as polyphenols, chalcones, and chlorogenic acids [12]. These compounds are known for their antimicrobial, antioxidant, and anti-inflammatory properties [13,14,15,16]. The valorization of such waste streams aligns with the European Union’s bioeconomy strategy, which encourages the repurposing of biomass for high-value applications [17,18].

This study aims to develop and evaluate aerosol formulations from CSS and HT phytoextracts for the disinfection of surfaces and airborne microbes in indoor environments. Through chemical characterization and microbiological efficacy testing using log reduction, this research explores the potential of these bio-based agents as sustainable alternatives to conventional chemical disinfectants. The investigation also integrates food production waste valorization, advanced spectroscopic analysis, and real-scale-up hygiene testing into a multidisciplinary framework for sustainable bio-disinfection technology.

## 2. Materials and Methods

### 2.1. Chemicals and Reagents

The methanol (Carl Roth, Karlsruhe, Germany), commercially available agar from Merck (Molsheim, France), Lysogeny broth agar (LB agar), lysogeny broth media (LB media) and neutralizing solutions were prepared at the microbiology laboratory of Constructor University.

### 2.2. Materials

Gollücke & Rothfos GmbH (Bremen, Germany) generously donated the coffee silverskin and the hot trub was obtained from the Bremen Beer Brewery Company, Germany (Becks GmbH & Co., KG, AnBev, Bremen, Germany). Moreover, this study used industrial disinfectant (ProPure Protect GmbH, Bremen, Germany), an air sampler (Merck Damstedt, Germany) for measuring airborne germs, a data logger (Merck, Damstedt, Germany) for measuring the temperature and humidity of the room, commercially available mold and yeast agar (HYCON^®^ Contact Slides SDX: Merck, Millipore S.A.S, 67,120 Molsheim, France), commercially available bacteria agar (HYCON^®^ Contact Slides TC: Merck, Millipore S.A.S, 67,120 Molsheim, France), and an aerosol device with a 300 µm nozzle size for spraying the formulated solution into the room.

#### 2.2.1. Extraction of Phytoextract from CSS and HT

In upscaling the phytoextract for the pilot bio-disinfection, 100 g of HT or CSS was accurately weighed using a balance, and 1 L of 70% methanol was added as the solvent. The mixture was sonicated for 20 min and then transferred to an overhead shaker for 12 h with stirring. The extracted solution was filtered through filter paper and evaporated using a rotary evaporator. The remaining concentrated solution was frozen at −80 °C and lyophilized to a dry powder. To recover all the ingredients, the experiment was repeated three times by adding fresh 70% MeOH to the filtrate, and all the extraction procedures were repeated. The final yield was determined by calculating the mean and standard deviation of the triplicates.

#### 2.2.2. Phytoextract Efficacy Test Strains

The test strains were selected in accordance with the requirements of the Association of Applied Hygiene’s disinfection commission certification for chemical disinfectant processes (Verbund für Angewandte Hygiene e.V. (VAH)). According to the field of interest of the disinfecting target, various microorganisms were selected. Both nonpathogenic and pathogenic bacteria, as well as both Gram-positive and Gram-negative organisms, were included for disinfection purposes. The selected strains included both pathogenic (*Listeria monocytogenes* and *Vibrio parahaemolyticus*) and nonpathogenic (*Escherichia coli* and *Bacillus subtilis*) species for Gram-positive and Gram-negative organisms, respectively. Their inclusion reflects their relevance to foodborne pathogens, such as *L. monocytogenes* and *V. parahaemolyticus*, and their environmental implication.

#### 2.2.3. Media and Solutions

All the culture media and solutions were prepared following standard operating procedures (SOPs). The LB media were prepared by dissolving 2 g of tryptone, 1 g of yeast extract, and 2 g of NaCl in 200 mL of MilliQ water to form the LB media. The agar was prepared by adding 3 g of agar per 200 mL of LB media.

#### 2.2.4. Bacteria Inoculum

The inoculum was prepared based on a previous study by our research group [19]. Briefly, two pathogenic organisms, *Listeria monocytogenes* (a Gram-positive bacterium) and *Vibrio parahaemolyticus* (a Gram-negative bacterium), were used. Two nonpathogenic microorganisms were also used, including *Bacillus subtilis* (a Gram-positive bacterium) and *Escherichia coli* (a Gram-negative bacterium), to cover a wide range of bacterial studies of the phytoextracts. All the bacterial strains were obtained from the frozen stock culture at the Constructor University, Bremen, Germany. The organisms were streaked on sterilized fresh LB agar and incubated overnight. A single colony of microorganisms was introduced into 5 mL of LB medium using a sterilized 1 mL pipette tip and incubated overnight on a shaker at 220 rpm at 37 °C or 28 °C, depending on the organism. The optical density (OD600 nm) of the bacterial solution was measured at 600 nm on the Jenway 6900 spectrophotometer. The obtained bacterial suspensions were diluted with LB medium to obtain a suspension with an OD600 nm corresponding to a cell concentration of 5 × 10^5^ CFU/mL.

#### 2.2.5. Water of Standardized Hardness (WSH)

The water of standardized hardness was prepared using two working solutions. Solution 1 was prepared by dissolving 19.8 mg/mL MgCl_2_ and 46.2 mg/mL CaCl_2_ in Milli-Q water, and solution 2 was prepared by dissolving 35 mg/mL NaHCO_3_ in Milli-Q water. The WSH was prepared by mixing 3 mL of solution 1 with 4 mL of solution 2. The WSH was used as the control solution for the efficacy testing of the model organisms.

#### 2.2.6. Neutralization Media for Efficacy Reaction

The neutralization solution was prepared as previously reported [20]. Briefly, the neutralization medium solution was used to stabilize the reaction between the test organism and the disinfectant when evaluating the efficacy of the phytoextract. The solution was prepared using varying percentages of Polysorbate 80, lecithin, histidine, and sodium thiosulfate.

#### 2.2.7. Efficacy Testing of CSS and HT Phytoextracts Against Model Bacteria

A suspension test to determine the efficacy of the disinfectants was used to determine the log reduction. Also, four model bacterial organisms were evaluated against the phytoextract solutions to determine the log reduction. The efficacy of the phytoextracts was tested against the following organisms: pathogenic organisms, *L. monocytogenes*, *Vibrio parahaemolyticus*, *Bacillus subtilis* and *Escherichia coli*. Briefly, to each 9.7 mL phytoextract or industrial disinfection solution, 0.2 mL of WSH and 0.1 mL of the inoculum were transferred and tested in a Falcon tube. The contents of the mixture in the Falcon tube were mixed and placed in a water bath at 20 ± 2 °C for 30 min and 60 min. Following the designated time interval, the mixture of 0.5 mL was transferred into 4.5 mL of a neutralization medium and thoroughly mixed to facilitate the neutralization reaction. The viable counts were determined after a neutralization time of 5 min ± 20 s plating on an agar plate. For the control and test condition experiments, 9.7 mL of WSH was used instead of the phytoextract solution. After 24 h of incubation, the colonies were counted and expressed as colony-forming units (CFUs) per milliliter (mL). The efficacy of the solution based on the log reduction factor of the phytoextract was computed by comparing the log reduction of the phytoextract with that of the control. The data analysis was performed using two-way ANOVA and Bonferroni’s multiple comparison tests, using GraphPad Prism 9.0.0, *n* = 3. The error bars denote the mean ± sd, *p*-value > 0.01 (*), *p*-value > 0.001 (**), *p*-value > 0.0001 (***), and *p*-value > 0.00001 (****) at a 95% confidence interval.

#### 2.2.8. Disinfection of a 20 m^3^ Room Using the Aerosol Technique for Airborne Microbes

An air sampler, the Merck 100 instrument, was used to collect 200 L of air on a commercially available agar plate at two positions for bacteria, yeast, and mold. The airborne and surface microbes were tested using commercially available standardized agar plates for yeast and mold (also used for the bacteria count). The agar plates were incubated for 48 h for bacteria and 96 h for yeast and mold. The colonies were counted after the growth time of each organism. The counted colonies contained a factor of 5 to one cubic meter of air. The measurement then provided the total counts of bacteria, yeast, and mold. The results were determined by comparing the number of colonies before and after disinfection.

#### 2.2.9. Disinfection of 20 m^3^ Room Using the Aerosol Technique for Surface Microbes

The room was designed for the positions where the samples were to be collected and indicated on the agar plates. The samples were collected before and after disinfection using the swiping and air collection methods. Agar plates containing bacteria, yeast, and mold were incubated for 48 and 96 h at 30 and 23 °C, respectively. The total bacterial count from the plates, as well as the yeast and mold, was measured using a 25 cm^2^ selective culture medium agar.

The temperature and humidity were controlled throughout the disinfection. The percentage of bacteria, yeast, and mold reduction was determined using the following formula:

(1) $$P=\left[\frac{A-B}{A}\right]\times 100\%$$

where *A* represents the microbial colony count before disinfection, and *B* is the microbial colony count after disinfection. *P* is the percentage of microbial reduction by the active substances.

Each disinfection assay was conducted in triplicate (*n* = 3) for all the tested surfaces (table, pump, shelf, and radiator) and air microbes (sampled from the radiator and shelf) for testing. The bacterial, yeast, and mold counts were recorded both before and after aerosol disinfection on each surface, as well as the air microbes under identical conditions. The same test was used for all the disinfection experiments (CSS, HT and PP) for both surfaces and air sampling positions. A significance threshold of *p* < 0.05 was used to determine statistical significance. These findings confirm the effectiveness of the disinfection procedure.

### 2.3. Chemical Characterization of CSS and HT Phytoextracts

Characterization was performed to elucidate the secondary metabolites responsible for the antimicrobial activities of the phytoextracts and to ascertain the safety of the extracts when used as disinfectants.

#### 2.3.1. LC-ESI-QTOF-MS Characterization of CSS and HT Phytoextracts

The obtained phytoextracts were characterized using the LC-MS/MS method described in our previous study, employing an Agilent 1260 UHPLC system (Karlsruhe, Germany) coupled with a Bruker Daltonics Impact QTOF MS (Bremen, Germany) equipped with an electrospray ionization (ESI) source operating in negative ionization mode [12,19].

#### 2.3.2. NMR Spectral Measurements of the Phytoextracts

NMR spectroscopy was performed as previously reported [21]. Briefly, the lyophilized CSS and HT phytoextract powder was dissolved in CDCl_3_ and filtered. The ^1^H NMR chemical shifts were reported in δ (ppm) and referenced to tetramethylsilane (TMS). The spectra were recorded using a Jeol 400 MHz NMR spectrometer. The ^1^H-NMR spectrum consisted of 128 scans. The baseline was corrected, and the resulting spectra were manually phased. The spectral data were viewed using mesReNova version 15.0, and the spectra for various classes of compounds were recorded, along with their corresponding chemical shifts. The predicted chemical shifts were generated using mesReNova software.

#### 2.3.3. FTIR Spectroscopy of the Phytoextracts

The FTIR spectra of the lyophilized HT and CSS phytoextract samples were obtained using a Nicolet-Avatar 370 FTIR spectrometer with KBr pellets in the range of 400–4000 cm^−1^. The spectra were recorded directly from the dry HT and CSS phytoextract samples without any solvent.

## 3. Results and Discussion

### 3.1. Extraction Yield of Upscaled HT and CSS for Aerosol Hygiene Disinfection Test

**Figure 1:**
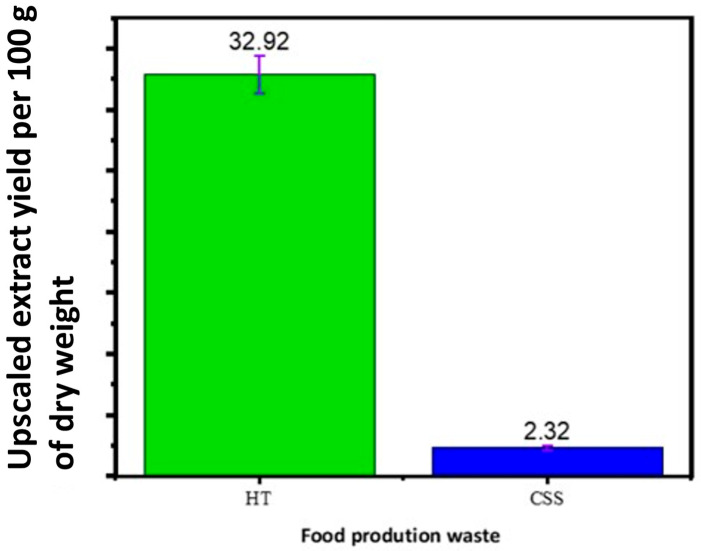
Upscaled extraction yield of hot trub (HT—green) and coffee silverskin (CSS—blue).

In the valorization process, determining the quantity of active ingredients in the waste phytoextracts is vital before recommending their industrial application. This study carefully upscaled HT and CSS to monitor the extraction yields. The HT phytoextract produced 32.92 g and the CSS 2.32 g of extract per 100 g dry waste (Figure 1). The HT waste phytoextract (100 g) could disinfect a 20 m^3^ room, while the CSS required 1.5 kg of waste for the same effect. A minimum of 30 g of crude phytoextract was needed to kill airborne and surface microbes. These results demonstrate that food production waste phytoextracts can be sustainably upscaled and used effectively for large-scale disinfection.

### 3.2. Efficacy Testing of Food Production Waste Phytoextracts Against Pathogenic and Nonpathogenic Organisms

Listeriosis and cholera are two life-threatening diseases that require attention to prevent their occurrence. Typically caused by consuming *Listeria*- and *Vibrio*-contaminated food, addressing these issues effectively is paramount. Prevention requires a multifaceted approach involving strict adherence to hygiene, temperature control, quality sourcing, monitoring, education, and compliance. The use of sustainable hygiene applications has been explored in this work toward new formulations for microbial disease prevention.

**Figure 2:**
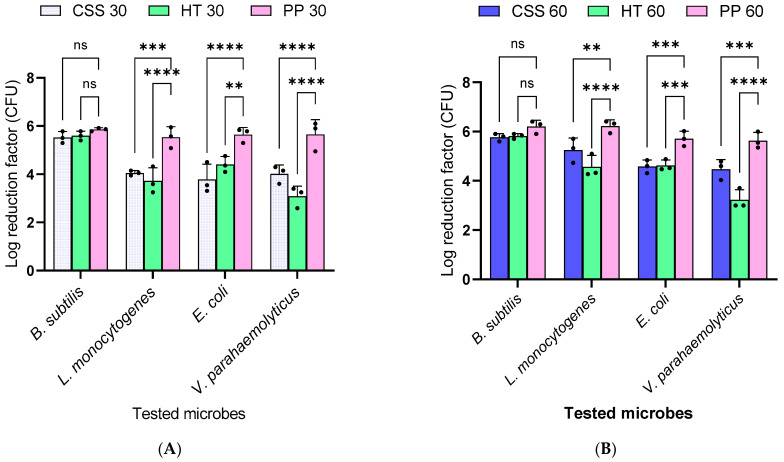
Efficacy of coffee silverskin (CSS) and hot trub (HT) phytoextracts and industrial disinfectant (PP) measured by the log reduction factor at (**A**) a 30-minute contact time (CSS30, HT30, PP30) and (**B**) 60-min contact time (CSS60, HT60, PP60). Data analysis was performed using two-way ANOVA and Bonferroni’s multiple comparison tests, using GraphPad Prism 9.0.0. *n* = 3. Error bars denote the mean ± sd, *p*-value > 0.001 (**), *p*-value > 0.0001 (***), and *p*-value > 0.00001 (****) at a 95% confidence interval and ns is no significance.

As a contribution to alternative green disinfection formulation, coffee-based waste, CSS, and brewery waste, HT, were assessed. The efficacy of these phytoextracts against *Listeria monocytogenes* and *Vibrio parahaemolyticus*, as pathogenic organisms, and *Escherichia coli* (*E. coli*) and *Bacillus subtilis*, as nonpathogenic organisms (Figure 2), was evaluated using two contact times. A 30 min contact time of the coffee silverskin phytoextract (CSS 30) formulation with the organisms resulted in a log reduction of 5.53, 4.05, 3.78, and 4.02 for *Bacillus subtilis*, *Listeria monocytogenes*, *E. coli*, and *Vibrio parahaemolyticus*, respectively (Figure 2A). Similarly, 60 min of CSS phytoextract being in contact with organisms resulted in a log reduction of 5.77, 5.25, 4.58, and 4.47, respectively (Figure 2B). There was a high significant difference at 30 min compared to the control (***) against *Listeria monocytogenes* (Figure 2A). At 60 min, contact time with *Listeria monocytogenes* and CSS phytoextract led to a significant difference compared to the control (**) (Figure 2B). However, comparing *Listeria monocytogenes* and CSS phytoextract solution, 60 min revealed moderately lower significance (**) than 30 min (***).

At 30 min, a commercial disinfectant (PP) achieved log reductions of 5.86, 5.53, 5.64, and 5.65 for *Bacillus subtilis*, *Listeria monocytogenes*, *E. coli*, and *Vibrio parahaemolyticus*, respectively. At 60 min, the log reductions improved to 6.2, 6.22, 5.71, and 5.63. The coffee waste phytoextract showing comparable effectiveness to PP further suggests its potential as a sustainable alternative to synthetic disinfectants in the food industry.

Additionally, at 30 min, the brewery waste phytoextract (HT) achieved log reductions of 5.59, 3.72, 4.41, and 3.08, improving the disinfection to 5.81, 4.56, 4.62, and 3.23 at 60 min against *Bacillus subtilis*, *Listeria monocytogenes*, *E. coli*, and *Vibrio parahaemolyticus*, respectively. The HT phytoextract also demonstrated comparable disinfection effectiveness to the coffee waste and industrial disinfectants, thereby conforming to the EU requirement for disinfectant formulation.

The PP, HT, and CSS phytoextracts showed greater inhibitory effects on Gram-positive bacteria (*B. subtilis* and *L. monocytogenes*) than on Gram-negative organisms (*E. coli* and *Vibrio parahaemolyticus*) at both 30 and 60 min (Figure 2A,B). However, comparing individual organisms, *L. monocytogenes* exhibited a higher log reduction with PP compared to the CSS and HT at both 30 and 60 min. For *E. coli*, PP demonstrated higher killing potential followed by the HT and CSS phytoextracts at both time reaction points. *Vibrio parahaemolyticus* exhibited a higher log reduction with the CSS than with the HT phytoextracts but a significantly higher reduction with PP at both contact times. Furthermore, it is worth noting that the 60 min duration yielded a slightly higher log reduction compared to 30 min, possibly due to the prolonged exposure to the antimicrobial phytoextracts. The antimicrobial activities, as measured by the log reduction of the tested bacteria, establish the potency and comparable antimicrobial efficiency of the phytoextracts compared to the hydrogen peroxide-based disinfectant. Similarly, the efficacy of the phytoextracts is based on the bioactive compounds present in the phytoextracts, predominantly polyphenols, as demonstrated by the LC-ESI-QTOF-MS data. Additionally, the results are consistent with previously published reviews, suggesting phytochemicals as alternatives to chemical disinfectants based on the non-toxicity of the phytoextracts [9,10,22,23,24].

The preliminary cost considerations suggest that the use of industrial food waste, such as coffee silverskin and hot trub, provides a low-cost feedstock base, as these materials are typically discarded and available at little to no cost. The main economic inputs arise from solvent extraction, energy-intensive drying processes, and formulation development. Although the phytoextract yields vary (CSS: 2.32 g/100 g versus HT: 32.92 g/100 g), the demonstrated antimicrobial efficacy suggests that even small amounts of bioactive-rich phytoextract can be effective.

### 3.3. Aerosol Hygiene Disinfection of Room Surfaces and Airborne Microbes

Ensuring safety in food production environments is crucial for human health. Microbes thrive in environments with variations in the nutrients, moisture, and temperature. Chemical disinfectants, though effective, are costly and potentially harmful to consumers and the environment. This study compared two food production waste phytoextracts with hydrogen peroxide (H_2_O_2_) for surface and air disinfection. The results revealed that the plant-based phytoextracts performed comparably to the control (H_2_O_2_), indicating their potential as safer and more sustainable alternatives. Notably, this is the first study to investigate the large-scale validation of HT and CSS extracts for disinfection in a large room (20 m^3^).

#### 3.3.1. Surface Disinfection Using H_2_O_2_-Based, HT and CSS Phytoextracts

**Figure 3:**
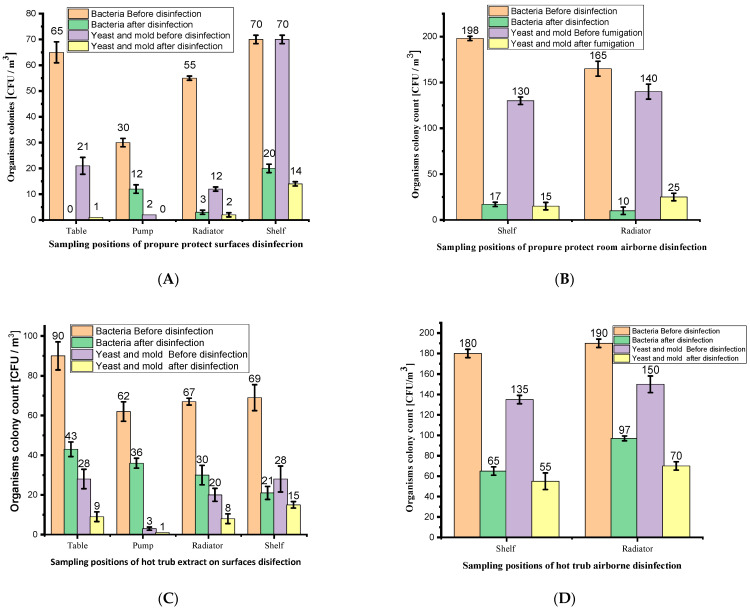

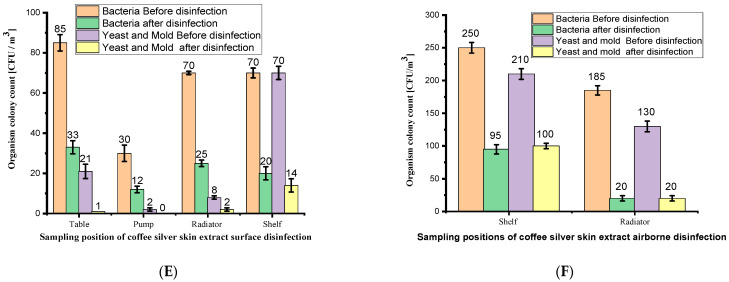
Aerosol hygiene disinfection of a 20 m^3^ room: surface and airborne microbial reduction using industrial disinfectant, hot trub, and coffee silverskin. (**A**,**C**,**E**) The surface disinfection results for PP, HT, and CSS, respectively; (**B**,**D**,**F**) show the corresponding airborne disinfection results. Data represents triplicate (*n* = 3) of the surface swabs and airborne sampling tests (colony-forming units ± standard deviation). Full numerical results are provided in Supplementary Figure S1. A *t*-test was also used for statistical analysis to determine the significance of the disinfection.

Four surfaces within the room (table, pump, radiator, and shelf) were selected for sampling to evaluate the disinfection effectiveness of the PP, 10% HT, and 10% CSS phytoextracts. The colony-forming units per cubic meter (CFU/m^3^) were measured before and after aerosol application of HT, CSS, and H_2_O_2_ in the 20 m^3^ room. On the table surface, the bacterial counts decreased from 65 ± 4 to 0 ± 0.0 for PP, from 90 ± 7 to 43 ± 3 for HT, and from 85 ± 4 to 33 ± 3 for CSS (Figure 3). The reduction represented 100%, 52%, and 62% for PP, HT, and CSS, respectively (Table 1). The outcome indicates that the CSS performed better than the HT phytoextracts. Similarly, the yeast and mold levels on the table surface decreased following aerosol disinfection with all the treatments: from 21 ± 3 to 1 ± 0 CFU/m^3^ with PP, from 28 ± 5 to 9 ± 2 with HT, and from 21 ± 4 to 1 ± 0 with CSS (Figure 3). The reduction effectiveness was indicated to be 68% for HT and 95% for both PP and CSS disinfection against yeast and mold (Table 1). Thus, the CSS aerosols showed similar antimicrobial efficacy to the industrial disinfectant against bacteria, yeast, and mold as the HT phytoextract.

Sampling of the microbial colonies before and after disinfection on the pump surface using PP, HT, and CSS showed bacterial reductions from 30 ± 2 to 12 ± 2 CFU/m^3^ (42%), 62 ± 4 to 36 ± 2 CFU/m^3^ (42%), and 30 ± 4 to 12 ± 2 CFU/m^3^ (60%), respectively (Figure 3). These percentages reflect the effectiveness of the bacterial reduction on the pump surface (Table 1). For yeast and mold, disinfection with PP, HT, and CSS resulted in reductions from 2 ± 0 to 0 ± 0 CFU/m^3^ (100%), 3 ± 0.8 to 1 ± 0 CFU/m^3^ (67%), and 2 ± 0 to 0 ± 0 CFU/m^3^ (100%). The percentages indicate that CSS and PP achieved complete yeast and mold reduction, outperforming HT on the pump surface (Table 1).

A radiator in the room was also evaluated, revealing bacterial colony reductions for PP, HT, and CSS of 55 ± 2 to 3 ± 0.8 CFU/m^3^ (95%), 67 ± 2 to 30 ± 5 CFU/m^3^ (55%), and 70 ± 0.8 to 25 ± 2 CFU/m^3^ (64%) (Figure 3). Table 1 indicates that PP had the highest antibacterial effectiveness, followed by CSS and then HT. For yeast and mold, the reductions on the radiator were 12 ± 0.8 to 2 ± 0.8 CFU/m^3^ (83%) for PP, 20 ± 3 to 8 ± 2.8 CFU/m^3^ (60%) for HT, and 8 ± 0.8 to 2 ± 0.8 CFU/m^3^ (100%) for CSS, with CSS performing the best (Table 1).

On the shelf surface, the fourth site sampled, the bacteria were reduced from 70 ± 2 to 20 ± 3 CFU/m^3^ (71%) for PP, 69 ± 7 to 21 ± 3 CFU/m^3^ (70%) for HT, and 70 ± 2 to 20 ± 2 CFU/m^3^ (71%) for CSS. The yeast and mold were reduced from 70 ± 3 to 14 ± 3 CFU/m^3^ (80%) with PP, 28 ± 7 to 15 ± 2 CFU/m^3^ (46%) with HT, and 70 ± 3 to 14 ± 3 CFU/m^3^ (80%) with CSS (Figure 3). The bacterial reduction was similar across all the treatments, while the yeast and mold reduction were equally effective for PP and CSS, with HT being less effective (Table 1).

Overall, the sampled surfaces demonstrated a significant microbial reduction when using the phytoextracts. These reductions exceeded those reported for other plant-based formulations [10]. This success may be attributed to synergistic antimicrobial mechanisms involving selected waste plant secondary metabolites such as polyphenols and lipids, which act on microbial cell walls or nucleic acid synthesis. Notably, the CSS and HT phytoextracts matched the efficacy of chemical disinfectants while supporting sustainability and resource circularity.

The surface disinfection tests revealed substantial reductions in the microbial loads for all three formulations: PP, CSS, and HT. Across the four tested surfaces (table, pump, radiator, and shelf), the bacterial counts before and after treatment were analyzed using paired t-tests. The statistical test confirmed that the reductions were significant for all the formulations before and after disinfection. For PP as the control, all the surfaces showed *p* < 0.005, with the radiator demonstrating the most important effect (*p* < 0.00012), indicating high efficacy across surfaces. Similarly, the CSS phytoextract formulation yielded significant reductions, with the *p*-values ranging from 0.00013 to 0.0098, being particularly effective on the radiator and shelf (*p* < 0.001). The HT formulation also resulted in statistically significant bacterial reductions on all the surfaces (*p* < 0.02), with the strongest effect observed on the radiator (*p* < 0.0089).

Regarding the surface disinfection against molds and yeasts, the PP and CSS formulations both demonstrated significant reductions on three out of four surfaces, while HT was only effective on the table surface (*p* < 0.0082). The CSS phytoextract achieved significance on the table, radiator, and shelf (all *p* < 0.02), while PP was effective on the same three surfaces. However, the pump surface consistently did not show statistically significant reductions in the fungal loads across all three formulations (all *p* > 0.07), suggesting that the surface material or microbial adhesion may reduce efficacy. Collectively, these results confirm that both byproduct-based formulations (CSS and HT) can achieve comparable antimicrobial surface performance to the commercial PP disinfectant, particularly against bacteria. The CSS showed better performance than the HT overall, especially against fungi, while the HT’s limitations appear most pronounced in terms of mold and yeast elimination.

#### 3.3.2. Aerosol Disinfection Against Airborne Microbes

The current study highlights the disinfection potential of waste phytoextracts, which achieved nearly the same reduction in bacteria, yeast, and mold as the chemical disinfectant. This effectiveness was attributed to bioactive metabolites such as chlorogenic acids (CGAs) from coffee waste (CSS) and terpenes from brewery waste (HT). These compounds retain their antimicrobial properties throughout the extraction and disinfection process [10,25].

Two locations in the room, the shelf and the radiator, were used for air sampling to assess the reductions in the bacteria, yeast, and mold. On the shelf, the colony-forming units per cubic meter (CFU/m^3^) decreased from 198 ± 2 to 17 ± 2 (91%) with PP, from 180 ± 4 to 65 ± 4 (64%) with HT, and from 250 ± 8 to 95 ± 7 (62%) with CSS, indicating bacterial disinfection efficiency. The mold and yeast reductions were 130 ± 4 to 15 ± 4 (88%) with PP, 135 ± 4 to 55 ± 8 (59%) with HT, and 210 ± 8 to 100 ± 4 (52%) with CSS (Figure 3), with the disinfection effectiveness ranked as PP > HT > CSS (Table 1).

On the radiator, the bacterial counts were reduced from 165 ± 8 to 10 ± 4 (94%) with PP, from 190 ± 4 to 97 ± 2 (49%) with HT, and from 185 ± 7 to 20 ± 4 (85%) with CSS. The mold and yeast decreased from 140 ± 8 to 25 ± 4 (82%) with PP, 150 ± 8 to 70 ± 4 (53%) with HT, and 130 ± 8 to 20 ± 4 (85%) with CSS (Figure 3). The bacterial disinfection effectiveness in the air was in the order PP > CSS > HT (Table 1).

Overall, the similar microbial reductions across different room positions confirm the effectiveness of the aerosol-based phytoextract disinfectants. The retained bioactivity of the phytoextracts following disinfection supports their reliability. Compared to past studies using surface-wiping methods, these aerosol treatments were more effective [10]. Both the HT and CSS aerosols demonstrate strong potential for large-scale disinfection use in food production, healthcare, and public spaces due to their eco-friendly natural bioactive compound contents.

In the aerosol disinfection test based on the statistical analysis, all the formulations demonstrated significant reductions in airborne bacteria and fungi on both the shelf and radiator. The PP formulation showed high statistical significance, with *p* < 0.00008 and 0.00242 for bacterial disinfection and *p* < 0.00063 and 0.00438 for mold and yeast, respectively. The CSS extract achieved similar success, with *p* < 0.0013 on all the surfaces for both bacteria and fungi. Notably, the CSS was highly effective in terms of the airborne yeast and mold reduction, with *p* < 0.00069 and 0.00479, comparable to the control (PP). The HT phytoextract also performed well, achieving significant reductions in bacterial and fungal growth on both test surfaces, including identical values of *p* < 0.0013 for airborne mold and yeast on the shelf and radiator surfaces.

These findings support the hypothesis that natural phytoextracts, when aerosolized through electrostatic methods, can significantly reduce both surface and airborne microbial loads. The high statistical strength of the results, particularly for the CSS, reinforces its potential as a scalable, environmentally sustainable disinfectant for enclosed indoor settings.

Although the concentration of the industrial disinfectant is lower than that of the two phytoextracts, future work on isolating the bioactive compounds from HT and CSS is recommended. Isolation and further testing could improve the activity of the phytoextract disinfection. While this study demonstrates the potential of the CSS and HT phytoextracts for large-scale aerosol disinfection, some of these challenges need to be overcome for wider industrial adoption. Optimization through purification or concentration of the active compounds can reduce the volumes required. Secondly, the long-term storage stability and microbial shelf-life efficacy of the crude phytoextracts should be tested. Future research should focus on formulation stabilization and standardization of the phytoextract strength. Finally, regulatory approval pathways and real-world pilot testing in clinical or food industry settings will be key steps toward commercialization.

#### 3.3.3. Monitoring Room Condition During Disinfection

The temperature, humidity, and dew point are critical parameters to monitor during aerosol disinfection, as they significantly affect the liquid-to-gas phase transition. Table 2 presents the room conditions during the aerosol disinfection. These factors can influence the size and distribution of mist droplets, potentially causing instability in aerosol dispersion. Proper moisture control is also crucial for preventing microbial growth and surface wetness. Combining phytoextracts with aerosol hygiene technology offers the potential to produce finer mist particles, enhancing the distribution throughout the room. The aerosol levels observed in this study were consistent with those reported in disinfectant formulations, effectively reaching all the areas without causing microbial growth [24].

### 3.4. FTIR Characterization of HT and CSS Extracts

**Figure 4:**
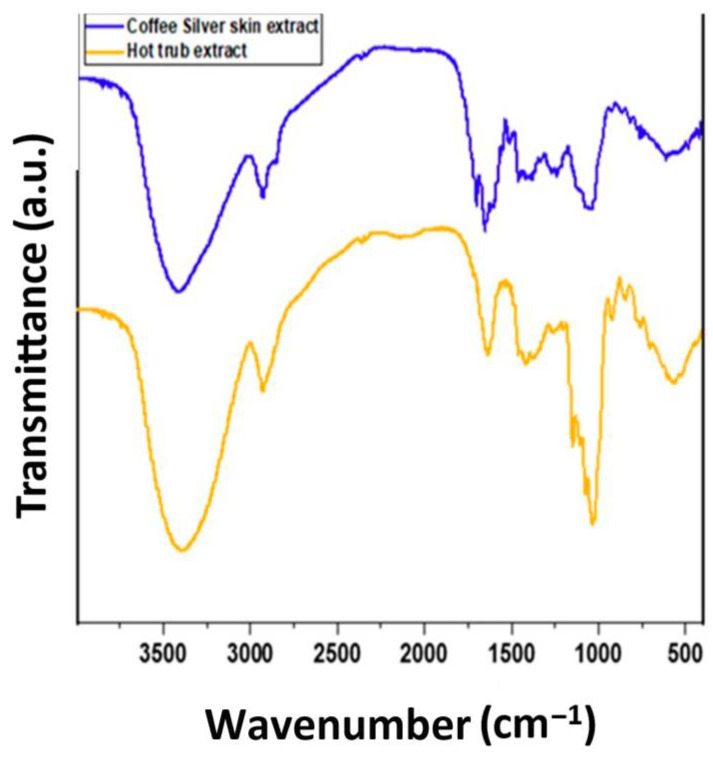
FTIR of the coffee silverskin and hot trub phytoextracts.

FTIR analysis was conducted to identify the functional groups present in both the HT and CSS extracts (Figure 4). The common features of both phytoextracts included broad bands around ~3280–3310 cm^−1^ (O–H stretching), peaks near 2925–2854 cm^−1^ (C–H aliphatic stretching), and strong signals around 1600–1700 cm^−1^ corresponding to aromatic C=C and C=O groups [26,27].

The CSS exhibited a prominent ester carbonyl band at 1724 cm^−1^, characteristic of chlorogenic acids, and aromatic ring vibrations around 1600 cm^−1^, suggesting a high polyphenolic content. The HT showed distinct bands at ~1440 cm^−1^ and ~1660 cm^−1^ [28], attributed to chalcones and bitter acids such as humulones and lupulones, which are typical of hop-derived residues (Figure 4). These differences highlight the specific chemical fingerprints of each extract and their potential bioactive roles.

### 3.5. H-NMR Characterization of HT and CSS Phytoextracts

**Figure 5:**
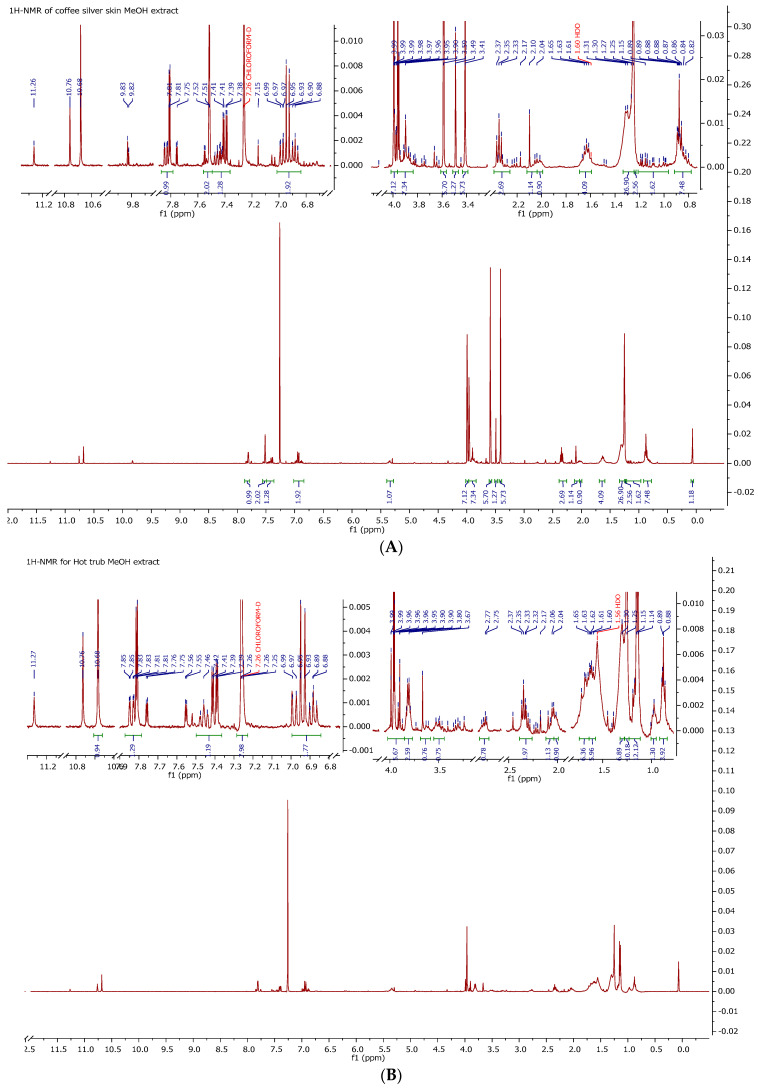
^1^H-NMR spectra of (**A**) coffee silverskin extract and (**B**) hot trub extract, displayed as the intensity versus chemical shift in ppm (400 MHz, using chloroform-d as the solvent).

The ^1^H-NMR spectra further confirmed the presence of bioactive compounds in the phytoextracts. For the CSS, major signals appeared in the aromatic region (6.2–7.6 ppm) [29,30], consistent with chlorogenic acids and related phenolic structures (Figure 5A). These resonances correspond to the FTIR observations of aromatic and ester groups [31].

The ^1^H NMR spectra revealed aliphatic multiplets at ~1.5–1.6 ppm, associated with methyl groups from bitter acids, along with distinct aromatic proton signals between 7.0 and 7.8 ppm. These spectra are indicative of chalcones and hop-derived polyphenols (Figure 5B). The complementary spectral profiles reinforce the unique composition of each phytoextract and support their respective antimicrobial characteristics.

Table 3 indicates the main FTIR and ^1^H-NMR spectral markers observed in the CSS and HT phytoextracts. Only compound-specific bands and chemical shifts relevant to distinguishing the bioactive compounds are listed. The data support the presence of chlorogenic acids in the CSS, and bitter acids and chalcones in the HT. Additional details regarding the chemical shifts and ^1^H NMR results are provided in the Supplementary Materials for CSS (Table S3A) and HT (Table S3B).

### 3.6. LC-ESI-QTOF-MS Characterization of CSS and HT Phytoextracts

Emerging fields of application, such as the hygiene technology industry, where the synergy of crude phytoextracts can function as sustainable disinfectants, have been established based on the antimicrobial potential of these phytoextracts. The characterization was based on a critical database search coupled with the literature comparison and fragmentation pattern of the compounds from the Brucker DataAnlysis 4.2. The profiling of the secondary metabolites was based on database matching, as previously described [19].

**Figure 6:**
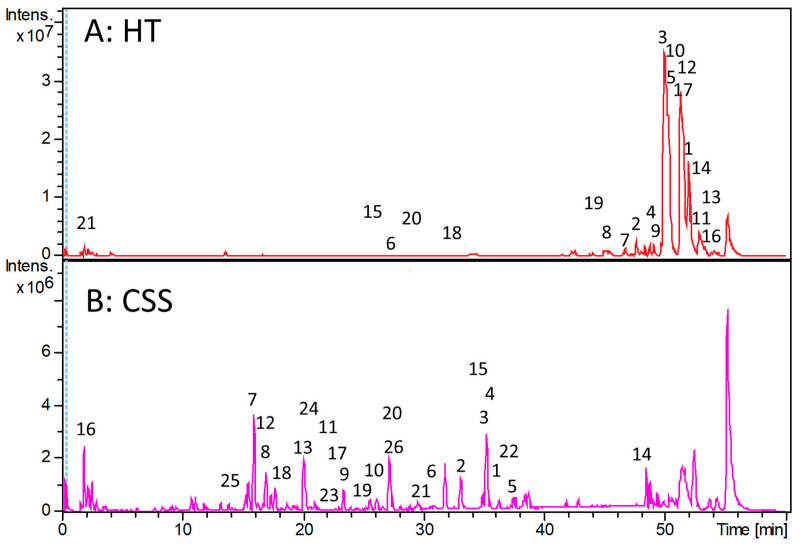
Chromatogram of the LC-ESI-QTOF-MS characterization of the secondary metabolites of the (**A**) HT and (**B**) CSS phytoextracts.

Characterization of the active ingredients in a new formulation is important to ascertain the safety of the final product. The total number of compounds identified in the HT phytoextract was 21 (Figure 6A). The detailed characterization of the compounds is presented in the Supplementary Materials (Table S2), with several matches to previously reported compounds. The most predominant compounds are α- and β-acids of hop, which is expected due to the partial insolubility of bitter acids during beer production [12]. It has been established that the α-acid present in beer production typically absorbs yeast cells, trub, and other materials, causing them to undergo various chemical changes and form several compounds [32]. Most of the phytoextract compounds, such as xanthohumol, humulone, and lupulone (Table S2), identified in the HT phytoextract have exhibited bioactivity against bacteria, including *Staphylococci*, in previous studies [33]. These compounds are predominantly chalcones, and there is well-established evidence of their biological potential, such as antimicrobial, antioxidant, anti-inflammatory, antifungal, and anti-HIV-1 activities [34].

Several derivatives of chlorogenic acids were identified in the CSS phytoextract, including 3-caffeoylquinic acid, 5-caffeoylquinic acid, 3,5-dicaffeoylquinic acid, 3,4-dicaffeoylquinic acid, and 4,5-dicaffeoylquinic acid, which are consistent with compounds previously reported [19]. A total of 26 compounds were profiled in the CSS phytoextract, with detailed information provided in Figure 6B and Supplementary Table S1. Characterization confirmed that the extraction process preserved the bioactive compounds, as evidenced by the bioactivity and disinfection effectiveness of the formulation.

**Figure 7:**
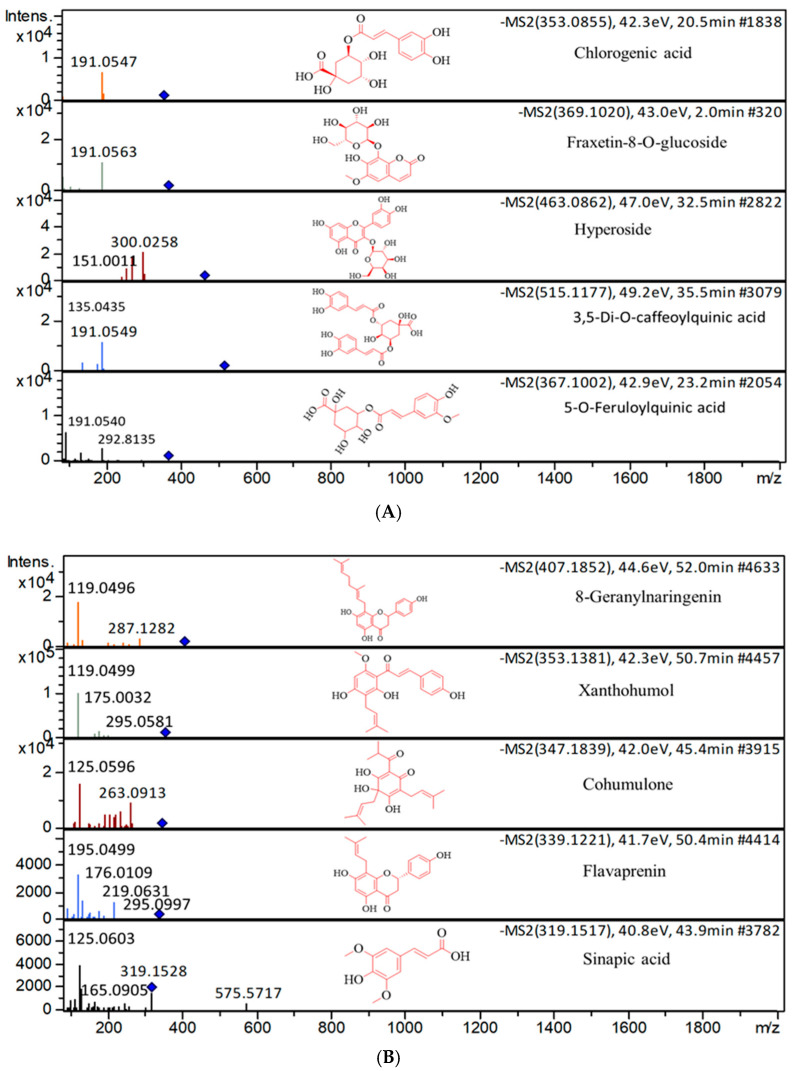
Extracted ion chromatogram of selected structures of bioactive phytoextracts from (**A**) CSS and (**B**) HT.

Plant secondary metabolites rely on the synergistic antimicrobial activities of multiple bioactive compounds working together to inhibit a broad range of microbes. The characterized CSS and HT phytoextracts revealed many of these established compounds, with five selected compounds highlighted from the CSS phytoextract (Figure 7A) and the HT phytoextract (Figure 7B).

## 4. Conclusions

The electrostatic aerosol spraying of antimicrobial agents from HT and CSS phytoextracts was shown to be highly effective against both pathogenic and nonpathogenic organisms, especially *Listeria monocytogenes* and *Vibrio parahaemolyticus*. The performance of the phytoextracts is comparable, with a significance level (*p* ≤ 0.05) similar to that of a hydrogen peroxide-based industrial disinfectant. This emphasizes the importance of developing natural-product-based disinfectants for combating foodborne pathogens, as well as sustainable aerosol label products. Practical tests of the disinfectant revealed substantial reductions in the bacteria, yeast, and mold on the surfaces and in the air of up to 100% within a 20 m^3^ room. Temperature and humidity monitoring indicated consistent levels throughout the aerosol disinfection, ensuring uniform aerosol distribution. LC-MS/MS, FTIR, and ^1^H-NMR spectroscopic chemical analysis identified chlorogenic acids (CQAs) as the major bioactive compounds in coffee silverskin, and prenylated compounds and chalcones as the key antimicrobial components in hot trub. The CSS and HT phytoextracts showed strong antimicrobial activity, significantly reducing the airborne and surface microbes. Future research should focus on isolating and concentrating the active ingredients, such as chlorogenic acids and chalcones, to enhance the efficacy and minimize the raw material usage. The results support the development of targeted formulations for food-processing, hospital, and agricultural environment applications. Given their effectiveness against Gram-positive bacteria such as *L. monocytogenes* and *B. subtilis*, these phytoextracts may be optimized for crop protection. Further studies should assess the persistence, toxicity, and compatibility with automated delivery systems.

## Figures and Tables

**Table 1:** Surface and airborne disinfection efficiency in a 20 m^3^ room by percentage.

Disinfectant/Formulation Microbes	Swiping Surfaces Reduction (%)				Airborne Reduction (%)	
Sampling positions	Table	Pump	Radiator	Shelf	Shelf	Radiator
H_2_O_2_-based						
Bacterial	100	42	95	71	91	94
Mold and yeast	95	100	83	80	88	82
HT phytoextract						
Bacterial	52	42	55	70	64	49
Mold and yeast	68	67	60	46	59	53
CSS phytoextract						
Bacterial	61	60	64	71	62	89
Mold and yeast	95	100	100	80	52	85

**Table 2:** Temperature, humidity, and dew point over the aerosol disinfection period. °C is degree Celsius and %rH is relative humidity in percentage.

Product	Room Condition	Minimum	Average	Maximum
H_2_O_2_-based disinfectant	Temperature (°C)	24.4	26.5	27.7
	Humidity (%rH)	38.4	50.1	60.2
	Dewpoint (°C)	10.4	15.1	18.7
HT Phytoextract	Temperature (°C)	24.8	26.0	27.0
	Humidity (%rH)	36.4	48.1	60.1
	Dewpoint (°C)	9.0	13.9	17.9
CSS Phytoextract	Temperature (°C)	21.6	23.0	26.4
	Humidity (%rH)	36.5	45.9	60.3
	Dewpoint (°C)	10.1	14.5	18.3

**Table 3:** Summary of the FTIR and ^1^H-NMR spectral markers for the CSS and HT phytoextracts, along with their functional interpretations.

Spectral Technique	Phytoextract	Key Markers	Functional Interpretation
FTIR	Coffee Silverskin	Chlorogenic acids (C=O ester stretch ~1724 cm^−1^, aromatic C=C ~1600 cm^−1^)	Supports the antioxidant and antimicrobial function of polyphenols
FTIR	Hot Trub	Chalcones (C=C conjugation ~1660 cm^−1^, aromatic C=C ~1600 cm^−1^)	Indicative of antimicrobial chalcone structures
FTIR	Hot Trub	Bitter acids (C–H bending ~1440 cm^−1^, phenolic O–H stretch)	Linked to hop-derived bitter acids with antimicrobial effects
^1^H-NMR	Coffee Silverskin	Chlorogenic acids (aromatic protons at 6.2–7.6 ppm)	Correlates with polyphenolic activity in bacterial membrane disruption
^1^H-NMR	Hot Trub	Aliphatic protons from bitter acids (multiplet at ~1.5–1.6 ppm)	Markers for hydrophobic antimicrobial components
^1^H-NMR	Hot Trub	Aromatic ring protons of chalcones (7.0–7.8 ppm)	Confirms the presence of aromatic antimicrobial metabolites

## Data Availability

The original contributions presented in the study are included in the article, further inquiries can be directed to the corresponding author.

## Supplementary Materials

The following supporting information can be downloaded at https://www.mdpi.com/article/10.3390/foods14142496/s1, Table S1: Profiling of the CSS compounds by LC-QTOF-MS; Table S2: Profiling of the HT compounds by LC-QTOF-MS; Table S3: Automatic integrated and predicted chemical shift of the ^1^H-NMR analysis of thecrude phytoextract using MestRenova v.14.2.3 software: (A) CSS and (B) HT; Figure S1: Details of the CSS and HT phytoextract disinfection efficacy as a percentage and the industrial disinfectant from ProPure Protect GmbH.
