# Supplementary material for: Development of Hot Trub and Coffee Silverskin Phytoextracts for Sustainable Aerosol Disinfectant Application
Source: Foods. 2025 Jul 16;14(14):2496. doi: 10.3390/foods14142496 (PMC12296017; doi:10.3390/foods14142496)
Supplement: Supplementary file 1 [file foods-14-02496-s001.zip › foods-3674136-supplementary.pdf]

foods

Supplementary Materials

Table S1. CSS.

#	RT/ min	[M-H]	Err	Meas. *m/z*	Theo. *m/z*	Compound	Base Peak ion	other fragments ions			
1	36.3	$C_{21}H_{19}O_{11}$	4.0	447.0915	447.0933	Kaempferol-3-O-glucoside	227.0333	255.0283	284.0310	256.0325	96.9590
2	32.5	$C_{21}H_{19}O_{12}$	4.4	463.0862	463.0882	Hyperoside	300.0258	271.0236	255.0292	301.0312	272.0269
3	34.7	$C_{25}H_{23}O_{12}$	5.4	515.1167	515.1195	3,4-Di-O-caffeoylquinic acid	173.0446	191.0547	135.0452	179.0330	161.0226
4	35.5	$C_{25}H_{23}O_{12}$	3.6	515.1177	515.1195	3,5-Di-O-caffeoylquinic acid	191.0549	135.0436	179.0344	136.0472	192.0574
5	37.9	$C_{25}H_{23}O_{12}$	5.4	515.1167	515.1195	4,5-Di-O-caffeoylquinic acid	173.0443	179.0344	135.0439	191.0552	93.0336
6	31.4	$C_{34}H_{25}O_{11}$	-4.1	609.1428	609.1402	Rutin	300.0255	301.0310	271.0242	255.0284	302.0355
7	15.8	$C_{16}H_{17}O_{9}$	4.7	353.0851	353.0878	5-cafeoyl quinic acid	191.0542	85.0289	87.0071	93.0327	192.0589
8	17.5	$C_{16}H_{17}O_{9}$	3.3	353.0856	353.0878	3-cafeoyl quinic acid	135.0434	93.0331	191.0543	173.0422	136.0497
9	23.2	$C_{17}H_{19}O_{9}$	4.5	367.1003	367.1003	5-O-Feruloylquinic acid	191.0557	134.0353	93.0334	87.0088	173.0438
10	27.2	$C_{17}H_{19}O_{9}$	3.9	367.1003	367.0976	4-O-Feruloylquinic acid	191.0538	85.0288	93.0339	192.0565	87.0089
11	21.9	$C_{17}H_{19}O_{9}$	4.7	367.1035	367.1006	3-O-Feruloylquinic acid	93.0334	134.0355	173.0444	111.0441	94.0363
12	15.8	$C_{39}H_{31}O_{13}$	0.3	707.1768	707.1770	4-cafeoyl quinic acid	191.0547	192.0581	161.0229	179.0336	93.0343
13	19.9	$C_{12}H_{15}O_{7}$	5.0	271.0799	271.0823	Arbutine	137.0250	149.0203	152.0466	123.0077	138.0293
14	47.5	$C_{15}H_{11}O_{5}$	3.8	271.0602	271.0612	Naringenin	119.0490	93.0336	83.0139	107.0113	161.0650
15	34.5	$C_{15}H_{17}O_{8}$	5.2	325.0912	325.0929	p-Coumaric acid-O-hexoside	78.9593	292.8046	229.8419	308.8069	102.9474
16	2	$C_{13}H_{21}O_{12}$	5.1	369.102	369.1038	Fraxetin-8-O-glucoside	191.0563	85.0290	103.0030	87.0090	129.0189
17	23.2	$C_{17}H_{19}O_{9}$	4.7	367.1003	367.1035	Methyl 5-O-caffeoylquinate	191.0556	134.0353	93.0334	87.0088	173.0438
18	18.5	$C_{9}H_{7}O_{4}$	4.8	179.0334	179.0350	Calffeic acid	134.0362	135.0424	89.0394	106.0431	118.0379
19	25.1	$C_{16}H_{17}O_{8}$	5.0	337.0899	337.0829	Coumaroyl quinic acid (isomer)	191.0543	85.0286	93.0354	111.0440	618.7287
20	27.2	$C_{17}H_{19}O_{9}$	4.8	367.1009	367.1035	5-O-Feruloylquinic acid	191.0560	85.0293	93.0338	134.0335	127.0385
21	30.1	$C_{16}H_{13}O_{6}$	5.7	301.07	301.0718	Quercetin	151.0051	164.0108	108.0195	136.0195	135.0400
22	37.1	$C_{15}H_{11}O_{6}$	4.4	287.0537	287.0561	Dihydrokaempferol	125.0230	151.0065	133.0281	152.0089	149.9032
23	21.8	$C_{16}H_{17}O_{8}$	4.8	337.0899	337.0929	4-O-p-Coumaroylquinic acid	93.0335	173.0462	119.0486	292.8072	111.0442
24	20.5	$C_{16}H_{17}O_{9}$	4.8	353.0851	353.0878	Chlorogenic acid	191.0542	85.0289	87.0071	93.0327	192.0589
25	13.6	$C_{7}H_{13}O_{7}$	3.2	209.066	209.0667	1,3,7-trimethyluric acid	137.0222	78.9583	179.0168	124.0505	80.0015
26	27.1	$C_{17}H_{19}O_{9}$	5.4	367.1004	367.1034	5-O-Feruloylquinic acid	191.0537	85.0288	93.0339	192.0565	87.0089

Table S2. HT.

#	RT/ min	[M-H]	Err	Meas. *m/z*	Theo. *m/z*	Compound	Base Peak ion		other fragments ions			
1	52	$C_{25}H_{27}O_{5}$	2.7	407.1853	407.1865	8-Geranylnaringenin	119.0496	287.1283	133.0649	201.0543	93.0338	201.0183
2	48.7	$C_{18}H_{33}O_{5}$	5.3	329.2316	329.2347	Desdimethyl-octahydro-iso-cohumulone	139.1130	211.1329	171.0998	127.1125	172.1068	99.0780
3	50.4	$C_{20}H_{19}O_{5}$	4.6	339.1222	339.1238	Flavaprenin	119.0498	133.0652	219.0630	93.0333	176.0108	151.0758

 https://doi.org/10.3390/xxxxx

4	49.1	$C_{21}H_{21}O_5$	5.1	353.1373	353.1394	Isoxanthohumol	119.0496	120.0529	133.0651	163.0025	175.0031	165.0904
5	50.7	$C_{21}H_{21}O_5$	3.5	353.1382	353.1384	Xanthohumol	119.0498	175.0032	163.0025	120.0526	203.0335	190.0636
6	27.3	$C_{26}H_{27}O_{14}$	3.8	563.1385	563.1406	Apigenin 6-C-pentosyl-8-C-hexoside	353.0654	383.0745	384.0734	365.0665	413.0918	296.0616
7	47.5	$C_{15}H_9O_5$	5.3	269.0439	269.055	Apigenin	117.0337	78.9583	149.0241	260.0873	159.0433	180.0546
8	45.4	$C_{20}H_{27}O_5$	3.8	347.184	347.1864	Cohumulone	125.0595	263.0913	233.1171	261.1107	193.0491	221.0446
9	49.9	$C_{19}H_{25}O_4$	4.2	317.1745	317.1758	Cohulupone	205.0863	133.0652	205.0499	152.0472	111.0446	233.0809
10	51.2	$C_{20}H_{27}O_4$	4.2	331.1901	331.1915	Hulupone	219.1014	125.0605	219.0651	191.0709	166.0629	247.0963
11	52.9	$C_{21}H_{29}O_5$	3.3	361.2008	361.2020	n-Cis -isohumulone	195.0657	125.0602	223.0602	196.0697	153.0187	163.0754
12	52.2	$C_{21}H_{29}O_5$	3.7	361.2007	361.2020	Iso-α-n/ad-humulone	219.1011	303.1591	245.0787	125.0602	219.0624	259.1727
13	54.1	$C_{26}H_{37}O_5$	4.8	429.2622	429.2646	Hydroxytricyclolupone /Hydroxytricycloadlupone	125.0599	245.1526	259.0972	176.0836	99.0803	78.9582
14	52.9	$C_{25}H_{35}O_5$ (-CH3)	3.2	415.2477	415.2490	Hydroxytricyclocolupone	111.0433	245.1485	148.0872	107.0486	125.0990	149.0980
15	26.1	$C_{27}H_{29}O_{15}$	5.1	593.1476	593.1512	Apigenin-C-hexoside-O-hexoside	311.0528	297.0374	282.0499	237.0897	283.0580	298.0407
16	52.9	$C_{21}H_{29}O_5$	3.6	361.2007	361.2020	cis-isoadhumulone	195.0657	125.0604	223.0601	196.0696	153.0188	163.0756
17	52	$C_{25}H_{35}O_5$	1.8	415.2482	415.2490	4-Hydroxycoluplone/Hydroxytricyclocolupone	111.0446	259.0946	181.0502	149.0968	203.1417	209.0455
18	32.4	$C_{28}H_{15}O_7$	-4.3	463.0857	463.0823	unkwon	271.0230	300.0236	301.0276	272.0284	243.0255	255.0288
19	43.9	$C_{18}H_{23}O_5$	4.1	319.1525	319.1551	deisopropyltricycloisohumulone	125.0607	319.1534	137.0963	165.0912	78.9597	554.4515
20	28.8	$C_{11}H_{11}O_5$	5.2	223.0594	223.0612	Sinapic acid	93.0331	94.0354	185.4320	121.0275		
21	1.8	$C_{12}H_{21}O_{11}$	3.9	341.1076	341.1089	Maltose	101.0238	161.8954	78.9558	103.0007		

Key: Err: error, RT: retention time, Min: minutes

Table S3. Automatic integrated and predicted chemical shift of 1H-NMR analysis of crude phyto-extract using MestRenova software A) CSS and B) HT.

S4A) 1H-NMR (400 MHz, Chloroform-d) δ 7.87 – 7.78 (m, 1H), 7.56 – 7.49 (m, 2H), 7.49 – 7.36 (m, 1H), 7.02 – 6.84 (m, 2H), 5.39 – 5.28 (m, 1H), 4.02 – 3.96 (m, 7H), 3.96 – 3.84 (m, 7H), 3.59 (s, 6H), 3.49 (s, 1H), 3.41 (s, 6H), 2.39 – 2.26 (m, 3H), 2.10 (s, 1H), 2.01 (s, 1H), 1.62 (q, J = 7.3 Hz, 4H), 1.21 – 0.96 (m, 2H), 0.92 – 0.78 (m, 7H), 0.07 (s, 1H).

S4B) 1H-NMR (400 MHz, Chloroform-d) δ 10.68 (s, 1H), 7.87 – 7.73 (m, 2H), 7.59 – 7.49 (m, 4H), 7.50 – 7.36 (m, 2H), 7.02 – 6.84 (m, 3H), 5.38 – 5.27 (m, 1H), 4.06 – 3.93 (m, 18H), 3.90 (s, 1H), 3.97 – 3.77 (m, 1H), 3.69 – 3.60 (m, 1H), 3.59 (s, 9H), 3.49 (s, 2H), 3.41 (s, 9H), 2.77 (t, J = 6.5 Hz, 1H), 2.35 (t, J = 7.5 Hz, 4H), 2.32 – 2.11 (m, 1H), 2.10 (s, 1H), 2.05 (d, J = 7.1 Hz, 1H), 2.01 (s, 2H), 1.62 (q, J = 7.3 Hz, 5H), 1.23 – 1.09 (m, 1H), 1.12 – 0.95 (m, 1H), 0.94 – 0.75 (m, 7H), 0.07 (s, 2H).

Swiping Surfaces	Bacterial Before disinfection	Bacterial after disinfection	Mold and Yeast Before disinfection	Mold and Yeast after disinfection
	CFU/m³			
Table	65±4	0±0	21±3	1±0
Pump	30±2	12±2	2±0	0±0
Radiator	55±0.8	3±0.8	12±0.8	20±0.8
Shelf	70±2	20±2	702	14±0.8

(**A**)

Air Sampling	Bacteria Before disinfection	Bacteria after disinfection	Mould and Yeast Before disinfection	Mold and Yeast after disinfectic
	CFU/m³			
Shelf	198±2	17±2	130±4	15±4
Radiator	165±8	10±4	140±8	25±4

(**B**)

Swiping Surfaces	Bacteria Before disinfection	Bacteria after disinfection	Mould and Yeast Before disinfection	Mould and Yeast after disinfection
	CFU/m³			
Table	90±7	43±4	28±5	9±2
Pump	62±4	36±2	3±0.8	1±0
Radiator	67±2	30±5	20±3	8±2
Shelf	69±7	21±3	28±7	15±2

(**C**)

Positions Airborne	Bacteria Before disinfection	Bacteria after disinfection	Mould and Yeast Before disinfection	Mould and Yeast after disinfection
	CFU/m³			
Shelf	180±4	65±4	135±4	55±8
Radiator	190±4	97±2	150±8	70±4

(**D**)

Swiping Surfaces	Bacteria Before disinfection	Bacteria after disinfection	Mould and Yeast Before disinfection	Mould and Yeast after disinfection
	CFU/m³			
Table	85±4	33±3	21±4	1±0
Pump	30±4	12±2	2±0.8	0±0
Radiator	70±0.8	25±2	8±0.8	2±0.8
Shelf	70±2	20±3	70±3	14±3

(**E**)

Positions Airborne	Bacteria Before disinfection	Bacteria after disinfection	Mould and Yeast Before disinfection	Mould and Yeast after disinfection
	CFU/m³			
Shelf	250±8	95±7	210±8	100±4
Radiator	185±7	20±4	130±8	20±4

(**F**)

Figure S1. Detailed information of disinfection results of surfaces and airborne microbes.
